# Serum cystatin C as a predictor of 90‐day mortality among patients admitted with complications of cirrhosis

**DOI:** 10.1002/jgh3.12543

**Published:** 2021-04-07

**Authors:** Anuchit Suksamai, Amnart Chaiprasert, Sakkarin Chirapongsathorn

**Affiliations:** ^1^ Division of Gastroenterology and Hepatology, Department of Medicine Phramongkutklao Hospital and College of Medicine, Royal Thai Army Bangkok Thailand; ^2^ Division of Nephrology, Department of Medicine Phramongkutklao Hospital and College of Medicine, Royal Thai Army Bangkok Thailand

**Keywords:** 90‐day mortality, biomarker, chronic liver disease, cirrhosis, creatinine, cystatin C, liver failure, model, model for end‐stage liver disease score, survival

## Abstract

**Background and Aim:**

Cystatin C (Cys) is not affected by age, sex, and muscle mass. We evaluated to compare the predictive performance of serum Cys level and model for end‐stage liver disease (MELD) score and developed a new model to predict 90‐day mortality among patients admitted with cirrhosis complications.

**Methods:**

A prospective cohort study was performed from December 2018 to December 2019. All cirrhotic patients admitted with acute decompensated liver cirrhosis or acute on chronic liver failure had laboratory values measured within 48 h of admission.

**Results:**

A cohort of 225 patients with cirrhosis was admitted during the study period. Sixty‐five patients were eligible for analysis. Twenty‐seven of these patients (41.4%) died within 90 days of follow‐up. The median of MELD score was 20.5 (15, 24). Serum Cys level of >1.45 mg/L had the highest 90‐day mortality prediction with the sensitivity and specificity of 66.7% and 68.4%, respectively. Cys and MELD scores were predictive of 90‐day mortality: Cys hazard ratio (HR) = 2.04 (95% CI 1.01–4.14, *P* = 0.048); MELD score HR = 1.01 (95% CI 0.51–2.01, *P* = 0.970). C‐statistic of Cys, MELD score, model for end‐stage liver disease‐cystatin C (MELD‐Cys) score, combined Cys with MELD‐Cys score to predict 90‐day mortality were 0.67, 0.58, 0.58, and 0.63, respectively. Adding Cys to the MELD score did not improve the predictive of 90‐day mortality.

**Conclusion:**

Serum Cys is superior to MELD score, and the new MELD‐Cys model is comparable to the MELD score in predicting mortality among patients with cirrhosis admitted with complications.

## Graphical abstract

Unlike creatinine, cystatin C (Cys) is not affected by age, sex, muscle mass. Serum Cys can use to predict mortality among patients with cirrhosis admitted with complications.

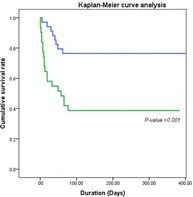

## Introduction

Hospitalized patients with cirrhosis are likely to increase the risk of acute kidney injury (AKI) for a variety of reasons, including diuretic therapy for ascites, abdominal paracentesis, gastrointestinal bleeding, infection, or hepatorenal syndrome (HRS), in which the mortality rate will increase.^1^, ^2^, ^3^

Model for end‐stage liver disease (MELD) score is widely used to determine the severity of patients with cirrhosis and to prioritize for liver transplants.^4^, ^5^ MELD and MELD sodium score can be used as the predictor of mortality in cirrhosis. However, they may not be good predictors of short‐term mortality when patients with cirrhosis have severe acute illness.^6^ The variables to be considered in MELD score include liver and kidney function.^7^, ^8^ Serum creatinine (SCr) is one of these variable parameters that is easily measurable and widely used as a marker of renal function. However, it could be highly influenced by muscle mass and protein intake. In patients with liver cirrhosis, discrepancies between SCr level and renal function can be accentuated by muscle wasting, protein‐calories malnutrition, and decreased hepatic synthesis. Moreover, several factors such as age, sex, and ethnicity can alter the SCr level.^1^ Ascites and peripheral edema can further decrease the level of SCr by widening the distribution of Cr in the body.^9^ Therefore, SCr levels used in the variables of MELD score may not be a good predictor of a mortality prediction in all patients with cirrhosis.

Cystatin C (Cys), which is a nonglycosylated 13 kDa protein, is a member of the cystatin superfamily of cysteine protease inhibitors.^10^ It is produced at a constant rate in all nucleated cells and freely crosses the glomerular membrane to be reabsorbed and metabolized in the renal proximal tubular cells, without extra renal elimination. Unlike Cr, Cys is not affected by age, sex, muscle mass, or liver function.^11^ Assessment of Cys levels could provide a better marker in the early detection of renal dysfunction than Cr levels.^12^, ^13^ Estimating kidney function in patients with liver cirrhosis is better performed by using serum Cys than Cr.^14^, ^15^ Cys levels could predict AKI and mortality within 90 days among patients with cirrhosis.^16^ Some factors that affected serum Cys levels such as receiving corticosteroid therapy, impaired thyroid function tests, HIV positive status, and malignancy.^17^, ^18^, ^19^, ^20^

Little evidence is available concerning the use of Cys as a predictor of mortality in hospitalized patients with cirrhosis. Our study aimed to compare the predictive performance of serum Cys level and MELD score and develop a new model to predict 90‐day mortality among patients admitted with complications of cirrhosis.

## Methods

### Subjects

We conducted a prospective trial from December 2018 to December 2019. Eligible individuals were aged between 18 and 80 years diagnosed with cirrhosis according to the combination of clinical, biochemical, imaging studies such as ultrasonography, computed tomography and magnetic resonance imaging, presence of varices, ascites, or liver biopsy, and admitted with acute decompensated liver cirrhosis or acute on chronic liver failure.

All consecutive hospitalized patients were screened and approached for enrollment by an internist and gastroenterologist at Phramongkutklao Hospital, Bangkok, Thailand. Exclusion criteria comprised patients with end‐stage renal disease receiving renal replacement therapy, pregnancy, hyperthyroidism, HIV status, and hepatocellular carcinoma (HCC) outside Milan criteria or others with active cancer. Information on medical history, age, sex, etiology of cirrhosis, and cause of admission were recorded from each patient. Laboratory evaluation including serum Cys, prothrombin time and international normalized ratio (PT/INR), albumin, bilirubin, aspartate aminotransferase (AST), alanine aminotransferase (ALT), sodium, blood urea nitrogen (BUN), and Cr were collected from all participants. Further biochemical values such as C‐reactive protein (CRP), procalcitonin, cortisol, lactate, and 25(OH)‐vitamin D were also evaluated. Child‐Pugh score (CTP), the MELD (MELD‐Na) scores, and glomerular filtration rate (GFR) according to chronic kidney disease epidemiology collaboration (CKD‐EPI) were performed to assess the liver and kidney functions.

### Study design

The eligible patients were evaluated by all laboratory values within 48 h of admission including serum Cys level. All enrolled patients were followed up for 90 days after discharge from the hospital or less if death had occurred earlier.

This study was approved by the institutional review board (IRB) and was registered in the national clinical registry; the registration number was TCTR20200205007. All patients completed written, informed consent forms to participate in the study. The study protocol conforms to the ethical guidelines of the 1975 Declaration of Helsinki as reflected in a priori approval by the institution's human research committee.

### Sample collection and method of measurement

Blood samples from each enrolled patient were collected for Cys and Cr at the same time within 48 h of admission. The laboratory staff were blinded to clinical data. Blood samples were stored at −20°C without freezing and thawing cycles to evaluate Cys levels. SCr levels were measured in the main biochemistry laboratory using the modified kinetic Jaffe reaction, which can measure SCr in jaundiced samples.

N Latex Cys assay by Siemens Healthineers Global, Erlangen Germany was used to determine Cys level. A latex‐enhanced turbidimetric inhibition immunoassay method was used to measure serum Cys level. For this assay, Cys in the sample underwent an agglutination reaction with anti‐human Cys in the sample. The serum Cys value was then calculated from a calibration curve in the absorbance value at a specific wavelength.

### Outcome measurement

The primary outcome was 90‐day mortality prediction after being admitted with cirrhosis complications.

The secondary outcomes were defined as described below.

- Cut‐off value of Cys level to predict 90‐day mortality.
- Predictive performance of combined MELD and Cys.

### Definitions

Cirrhosis^21^ is defined as the stage of chronic liver disease characterized by increasing portal pressure and impaired liver function according to combined clinical, biochemical, and imaging studies such as ultrasonography, computed tomography and magnetic resonance imaging, and the presence of varices, ascites, or liver biopsy.

Alcoholic cirrhosis^22^, ^23^ is diagnosed by documenting regular alcohol consumption of more than 20 g daily among women and 30 g daily among men together with the presence of clinical or biochemical evidence of cirrhosis without the presence of any other causes.

Acute decompensated liver cirrhosis^24^ is defined as the acute development of overt clinical signs including ascites, bleeding, encephalopathy, and jaundice.

HRS^25^ is defined by the International Ascites Club (ICA) as diagnosis of cirrhosis with ascites, diagnosis of AKI according to ICA‐AKI criteria, no improvement of Cr after at least 2 days with diuretic withdrawal and volume expansion with albumin 1 g/kg of body weight daily up to a maximum of 100 g/day, absence of shock, no current or recent treatment by nephrotoxic drugs, and absence of parenchymal kidney diseases indicated by proteinuria more than 500 mg/day, microhematuria (>50 red blood cells per high power field), or abnormal renal ultrasonography.

AKI^26^ is defined as any of the parameters described below.

1. Increase in SCr by >0.3 mg/dL within 48 h.
2. Increase in SCr to >1.5 times baseline, which was known or presumed to have occurred within the prior 7 days.
3. Urine volume < 0.5 mL/kg/h for 6 h.

Chronic kidney disease (CKD)^27^ progression is based on one or more of the parameters described below.

1. Decline in GFR category (>90 [G1], 60–89 [G2], 45–59 [G3a], 30–44 [G3b], 15–29 [G4], <15 [G5] mL/min/1.73 m^2^). A certain drop in estimated GFR (eGFR) was defined as a drop in GFR category accompanied by a 25% or greater drop in eGFR from baseline.
2. Rapid progression of GFR was defined as a sustained decline in eGFR of more than 5 mL/min/1.73 m^2^/year.
3. The confidence in assessing progression was increased with increasing number of SCr measurements and duration of follow‐up.

MELD score^28^ is calculated using the equation described below.

$$\text{MELD}\;\text{score}=9.57\times \log_{e}\left(\mathrm{Cr},\mathrm{mg}/\mathrm{dL}\right)+3.78\;\log_{e}\left(\text{bilirubin},\mathrm{mg}/\mathrm{dL}\right)+11.2\times \log_{e}\left(\mathrm{INR}\right)+6.43.$$

MELD‐Cys is calculated by substituting Cr within the equation with Cys level.

### Statistical analysis

Sample size calculation was determined from the trial of Chung et al.^16^ with a power of 0.9 and an alpha significance level of 0.05 (two‐sided) to predict the performance of serum Cys level with 90‐day mortality. The number of patients should total at least 65 patients.

Baseline clinical and biochemical data were compared using independent t test or Mann–Whitney *U*‐test, and Fisher's exact test for categorical variables, while continuous variables were compared using repeated anova test and chi‐square test with appropriate degrees of freedom. Continuous variables were presented as mean + SD for normal distribution data, median with interquartile range (IQR) for non‐normal distribution, while categorical variables were presented as percentage and frequency over the total available. Patient survival was calculated using the Kaplan–Meier method and differences between subgroups were compared using the log‐rank test. Multiple Cox proportional hazards regression was used to estimate the coefficient of Cys for the MELD‐Cystatin model. Receiver operating characteristic (ROC) analyses were applied to summarize discrimination with the area‐under‐the‐curve (AUC) statistic and 95% confidence interval (CI). Significance was established at a *P* value of <0.05. The analyses were performed using IBM SPSS Statistics for Windows, Version 22.0 (IBM Corp., Armonk, NY, USA).

## Results

### Study population

**Figure 1:**
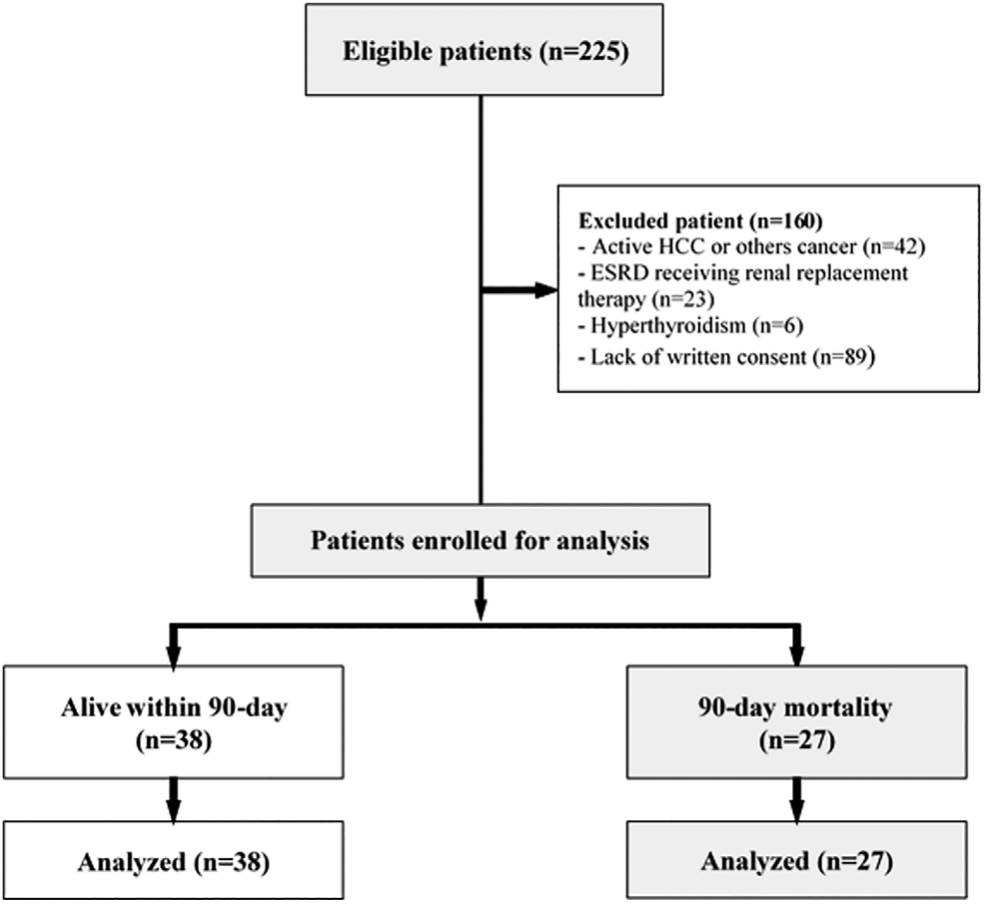
Protocol of study.

Two hundred and twenty‐five patients admitted with cirrhosis complications were screened from December 2018 to December 2019. One hundred and sixty patients were excluded, leaving 65 patients enrolled, as depicted in Figure 1. The reasons for excluding the screened patients were failing to meet the inclusion criteria and compatible with any of the exclusion criteria or the patient declined to participate.

Sixty‐five patients were eligible for the analysis. Twenty‐seven (41.4%) of these patients died within 90 days of follow‐up. Most of which were caused by infection, 18 of 27 patients (66.7%). spontaneous bacterial peritonitis (SBP) and primary bacteremia constituted the majority. Acute variceal bleeding and hepatic encephalopathy were present as the cause of death, 8 (29.6%) and 4 (14.8%) of 27 patients, respectively. The baseline demographic characteristics of the study population were similar between living and dead groups as shown in Table 1. The mean age of patients was 60 ± 12.8 years. The major cause of cirrhosis was alcohol (*n* = 42, 64.6%). The median of Child‐Pugh score was 10 (8, 11). Thirty‐three of these patients had Child‐Pugh score C (50.8%). The median of MELD score and MELD‐Na were 18 (14, 24) and 22 (17, 26.5), respectively. Comorbidity of the patients did not significantly differ between two groups. Diabetes was the most common comorbidity of the patients.

**Table 1:** Characteristic demographic data

Variables	Alive (*n* = 38)	Death (*n* = 27)	*P* value
Age (years)	58 ± 12.73	63 ± 12.56	0.094
Male	27 (71)	21 (77)	0.582
Etiology			
Alcohol	25 (65.7)	17 (63)	0.510
Hepatitis B virus	8 (21)	9 (33.3)	0.391
Hepatitis C virus	3 (7.9)	2 (7.4)	0.661
Nonalcoholic steatohepatitis	2 (5.2)	2 (7.4)	0.555
Autoimmune hepatitis	1 (2.6)	0	0.585
Child‐Pugh score	9 (7, 10)	10 (8, 12)	0.841
CTP A	2 (3.1)	1 (1.5)	0.333
CTP B	18 (27.7)	11 (16.9)	0.379
CTP C	18 (27.7)	15 (23.1)	0.454
MELD score	17.5 (13, 23.25)	20 (15, 27)	0.133
MELD‐Na score	21.5 (17–26.25)	22 (17, 32)	0.349
Underlying disease			
Diabetes	20 (52.6%)	9 (33.3%)	0.123
Hypertension	16 (42.1%)	9 (33.3%)	0.474
Dyslipidemia	11 (28.9%)	5 (18.5%)	0.336
Chronic kidney disease	4 (10.5%)	3 (11.1%)	0.940
Pulmonary disease	2 (5.2%)	1 (3.7%)	0.768
Coronary artery disease	0 (0%)	1 (3.7%)	0.199
Cause of admission			
GI bleeding	7 (18.4%)	8 (29.6%)	0.291
Hepatic encephalopathy	6 (15.7%)	4 (14.8%)	0.915
Refractory ascites	1 (12.5)	0	0.396
Spontaneous bacterial peritonitis	7 (18.4)	6 (22.2)	0.706
Primary bacteremia	18 (47.3)	12 (44.4)	0.816
Acute kidney injury	3 (7.9)	5 (18.5)	0.199
Biochemical value			
Interquartile change	1.51 (1.3–1.8)	1.45 (1.3–2)	0.671
Albumin (g/dL)	2.46 (2.1–3)	2.62 (2.3–2.8)	0.731
Bilirubin (mg/dL)	2.6 (1.5–9.9)	2.7 (1.8–9.7)	0.227
Aspartate aminotransferase (IU/L)	63.5 (44.2–98.7)	124 (56–212)	0.022
Alanine aminotransferase (IU/L)	29.5 (20.3–41.2)	38 (22–86)	0.022
Sodium (mEq/L)	134 (130–137)	135 (130–137)	0.800
BUN (mg/dL)	19.7 (10.5–28.3)	28.4 (17.7–37.8)	0.066
Creatinine (mg/dL)	1.04 (0.7–1.7)	1.5 (0.9–2.2)	0.078
Procalcitonin (ng/mL)	1.29 (0.2–2.5)	0.54 (0.2–2.6)	0.510
C‐reactive protein (ng/L)	23.5 (14.3–44.6)	26 (15.6–65)	0.560
Lactate (mmol/L)	2.33 (1.7–2.8)	2.8 (1.8–5.3)	0.050
25(OH)‐vitamin D (mg/dL)	22.07 (17–29.2)	20 (12–32)	0.860
Cortisol (μg/dL)	17.15 (10.1–22)	18 (13.6–24)	0.110

CTP, Child‐Turcotte‐Pugh; MELD, model for end‐stage liver disease.

### Biochemical values

The median of Cys level collected within 48 h of admission was significantly higher within the dead group compared with the living groups, 1.66 (1.02, 2.23) mg/L vs. 1.15 (0.89, 1.65); *P* = 0.026. Serum AST and ALT levels were also significantly higher within the death group compared with the living group, 124 (56, 212) vs. 63.5 (44.2, 98.7); *P* = 0.022 and 38 (22, 86) vs. 29.5 (20.3–41.2); *P* = 0.022. SCr in the death group was not significantly higher than that in the living group, 1.5 (0.9, 2.2) vs. 1.04 (0.7, 1.7) mg/dL; *P* = 0.078. Other biochemical values did not significantly differ between the two groups. The median of procalcitonin, CRP, lactate, 25(OH)‐vitamin D, and cortisol within dead and living groups were 0.54 (0.2, 2.6) vs. 1.29 (0.2, 2.5) ng/mL; *P* = 0.510, 26 (15.6, 65) vs. 23.5 (14.3, 44.6) mg/L; *P* = 0.560, 2.8 (1.8, 5.3) vs. 2.33 (1.7, 2.8) mmol/L; *P* = 0.050, 20 (12, 32) vs. 22.07 (17, 29.2) mg/dL; *P* = 0.860, 18 (13.6, 24) vs. 17.15 (10.1, 22) μg/dL; *P* = 0.110, respectively (Table 1).

### Factors associated with the 90‐day mortality

According to univariable Cox proportional hazards analysis, serum Cys (hazard ratio [HR] 2.056; 95% CI 1.02–4.12; *P* = 0.043) and age (HR 1.092; 95% CI 1.01–1.18; *P* = 0.048) were associated with the 90‐day mortality. However, only serum Cys was associated with the 90‐day mortality according to multivariable Cox proportional hazards analysis, HR 2.042; 95% CI 1.01–4.14; *P* = 0.048 (Table 2).

**Table 2:** Factors associated with 90‐day mortality

Variables	Univariate HR (95% CI)	*P* value	Multivariate HR (95% CI)	*P* value
Cystatin C	2.056 (1.02–4.12)	0.043	2.042 (1.01–4.14)	0.048
Age (years)	1.092 (1.01–1.18)	0.048	1.034 (0.99–1.08)	0.097
Gender, Male	0.400 (0.05–3.05)	0.377		
MELD score	1.013 (0.51–2.01)	0.970		
MELD‐Na score	0.958 (0.54–1.68)	0.883		
CTP score	1.926 (0.84–4.44)	0.124		
Biochemical value				
Albumin(g/dL)	0.790 (0.18–3.43)	0.753		
Bilirubin(mg/dL)	1.067 (0.84–1.36)	0.595		
Aspartate aminotransferase (IU/L)	1.003 (0.99–1.01)	0.586		
Alanine aminotransferase (IU/L)	1.013 (0.99–1.04)	0.336		
Interquartile change	0.277 (0.01–14.57)	0.526		
Sodium (mEq/L)	1.078 (0.88–1.31)	0.459		
BUN (mg/dL)	1.033 (0.92–1.15)	0.571		
Creatinine(mg/dL)	0.786 (0.05–12.68)	0.865		
Procalcitonin (ng/mL)	0.996 (0.77–1.28)	0.977		
C‐reactive protein (ng/L)	1.009 (0.99–1.03)	0.427		
Lactate (mmol/L)	0.953 (0.72–1.26)	0.742		
25(OH)‐vitamin D (mg/dL)	0.980 (0.9–1.06)	0.606		
Cortisol (μg/dL)	1.046 (0.94–1.16)	0.413		

CI, confidence interval; HR, hazard ratio; MELD, model for end‐stage liver disease.

### Predictive performance of 90‐day mortality with Cys

**Figure 2:**
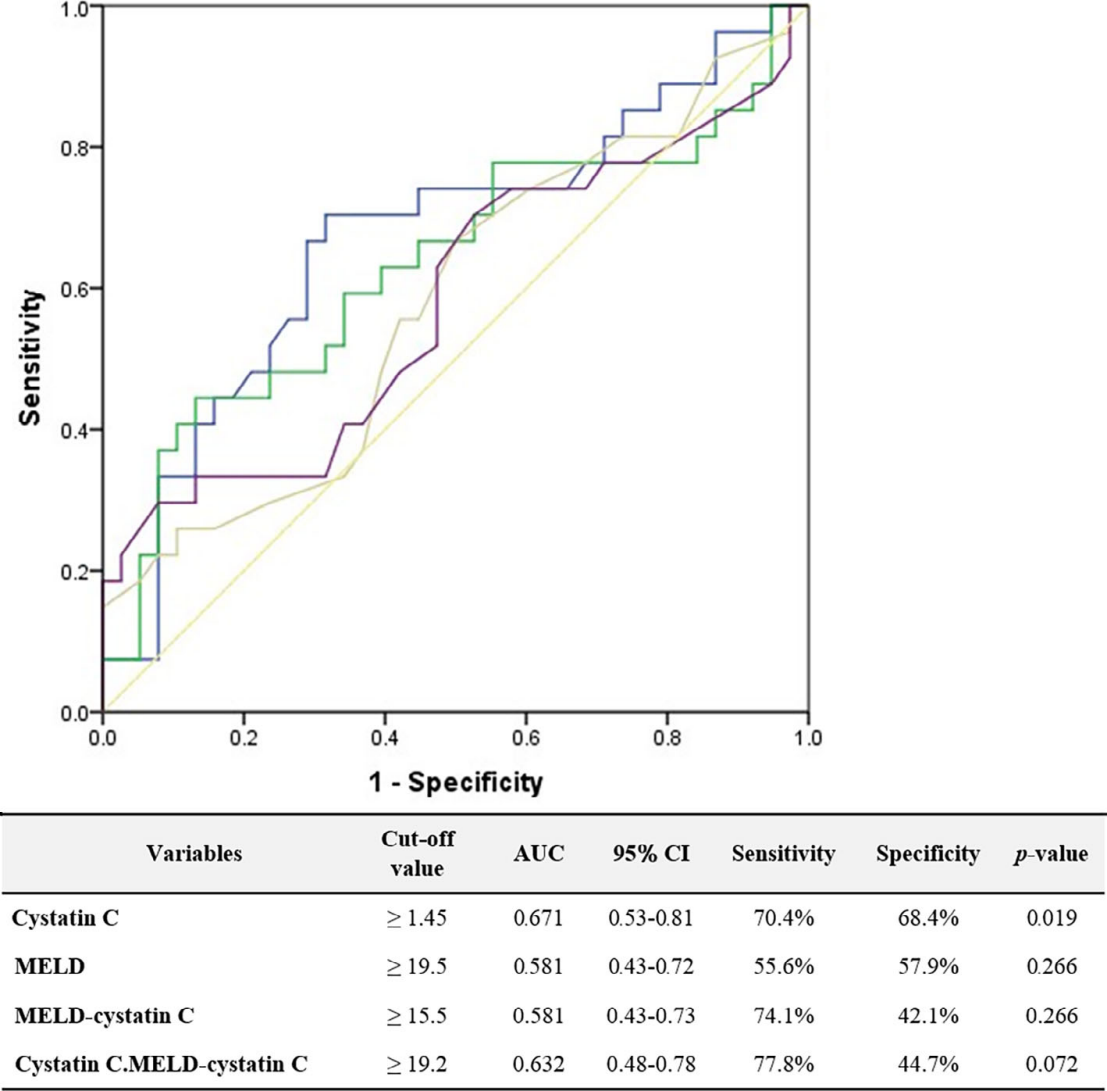
Receiver operating characteristic curve for predicting the 90‐day mortality. Source of the curve: 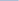, Cystatin C; 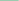, combine cys. MELD‐cys; 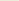, MELD; 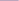, MELD‐Cys; 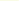, reference line. MELD, model for end‐stage liver disease.

The discriminative power of the scores in predicting 90‐day mortality was compared using ROC curve analysis. Cys could predict the 90‐day mortality with a cutoff value at 1.45 mg/L (AUC 0.671, 95% CI: 0.53–0.81; *P* = 0.019), providing a sensitivity of 70.4% and a specificity of 68.4%. MELD score and MELD‐Cys might predict the 90‐day mortality with a cutoff value at 19.5 (AUC 0.581, 95% CI: 0.43–0.72; *P* = 0.266), providing a sensitivity of 55.6% and a specificity of 57.9% and 15.5 (AUC 0.581, 95% CI: 0.43–0.73; *P* = 0.266), providing a sensitivity of 74.1% and a specificity of 42.1%, respectively. Combined use of both Cys and MELD‐Cys might predict the 90‐day mortality with a cutoff value at 19.2 (AUC 0.632, 95% CI: 0.48–0.78; *P* = 0.072), providing a sensitivity of 77.8% and a specificity of 44.7% (Fig. 2).

**Figure 3:**
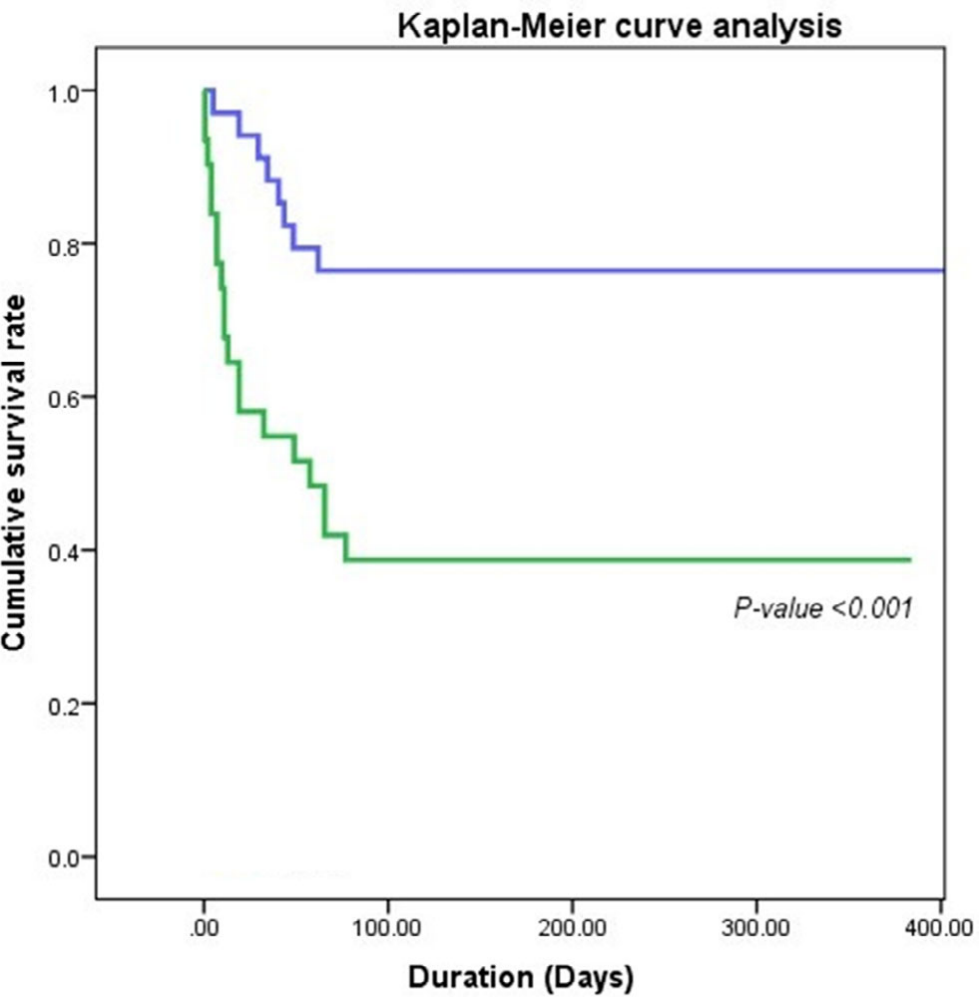
Kaplan–Meier survival in cirrhosis by selected Cystatin C level at cut‐off value >1.45 mg/L. Cystatin C (mg/L): 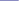, <1.45; 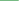, ≥1.45.

Patients having serum Cys level equal to or more than 1.45 mg/L exhibited significantly higher mortality than those not having, *P* = < 0.001 (Fig. 3).

### Discussions

Our study depicted five key findings. First, alcohol was the most common cause of cirrhosis. Second, infection was the major cause of death in hospitalized patients with cirrhosis. Third, high serum Cys is a risk factor of 90‐day mortality. Fourth, serum Cys level equal to or more than 1.45 mg/L could be used to predict 90‐day mortality among patients hospitalized with cirrhosis complications. Lastly, combined use of both Cys and MELD did not improve the predictivity of 90‐day mortality much.

Patients with cirrhosis were prone to develop AKI due to various conditions and were associated with increased mortality risk.^1^, ^2^, ^3^ Serum Cys performed better as an independent factor of renal injury than SCr in cirrhosis because of the unaffected ability of Cys regarding protein‐calories malnutrition, muscle mass, age, and sex.^12^ Using Cys showed better results in estimating kidney function than Cr among patients with cirrhosis.^14^ Moreover, serum Cys could predict 90‐day mortality among patients with cirrhosis and development of HRS type 1 among patients with cirrhotic ascites from one related study.^16^ This prospective single‐center study has contributed to evaluating the value of Cys in predicting 90‐day mortality among patients admitted with complication of cirrhosis. In our study, we included all patients with cirrhosis, not only patients with cirrhotic ascites.

In our study, the most common etiology of cirrhosis was alcohol (64%). Other etiology included alcohol (64%), chronic viral hepatitis (34%), NASH (6%), and autoimmunity (1.5%). The median of MELD score was 18 (14, 24). Similarly, the study of Finkenstedt et al.^29^ reported the etiology of cirrhosis included alcohol and NASH (58.7%), chronic viral hepatitis (25.6%), and cryptogenic (5.8%), and the median of MELD score was 12 (9–17). These findings supported the increasing prevalence of alcohol use disorder in Thailand and worldwide. Alcohol might be related to the worsening outcome by various pathophysiology but we did not explore who was active or abstinent alcohol drinking in our study. There was no statistical difference between the number of survival and non‐survival alcoholic cirrhosis who were admitted with acute decompensated cirrhosis in our study.

The 90‐day mortality in this study was 41.4%, and infection (66%) including SBP and primary bacteremia was the most common cause of admission and death among patients with cirrhosis. Acute variceal bleeding (29.6%) and hepatic encephalopathy (14.8%) were present as the cause of admission and death according to complication of portal hypertension. These findings were consistent with the study of Finkenstedt et al.,^29^ reporting that the main causes of death included multi‐organ failure with or without sepsis (59%), variceal or non‐variceal bleeding (19%), and hepatic decompensation (17%).

High serum Cys is a better independent factor for predicting 90‐day mortality with a hazard ratio of 2.042 (95% CI: 1.01–4.14) compared with SCr. Thereby, the greater sufficient sensitivity of serum Cys regarding kidney dysfunction than SCr is associated with poor outcome in decompensated liver cirrhosis. Our study showed consistent findings as in the recent study by Belcher JM, et al.,^30^ demonstrating a mortality prediction of Cys with a hazard ratio of 2.27 (95% CI: 1.07–4.85). However, the result of our study showed less predictive value than the study of Chung et al.,^16^ presenting a hazard ratio of 8 (95% CI: 2.16–29.64).

Serum Cys level at equal to or more than 1.45 mg/L depicted the best performance as a prediction of 90‐day mortality, providing a sensitivity of 79.4% and a specificity of 68.4%. This result was similar to the study of Chung et al.,^16^ showing a cutoff value of Cys >1.23 mg/L had a sensitivity of 66% and a specificity of 86% for predicting AKI and short‐term disease‐related mortality. Our findings could confirm the good precision of Cys as a mortality prediction among patients with cirrhosis.

The area under the curve of Cys significantly predicted the 90‐day mortality compared with other clinical models (AUC, 0.671; 95% CI: 0.48–0.78), *P* = 0.019. Combined use of both Cys and MELD‐Cys might predict the 90‐day mortality with AUC of 0.632; 95% CI: 0.48–0.78, *P* = 0.072, which is higher than MELD, AUC of 0.581; 95% CI: 0.43–0.72, *P* = 0.266, and MELD‐Cys, AUC of 0.581; 95% CI: 0.43–0.73, *P* = 0.266. Substituting Cr by Cys in the parameter of MELD score to predict mortality did not improve the performance much. A benefit of combined Cys and a Cys based MELD score may be that it would be less affected by muscle mass and sex among patients with cirrhosis.^11^ This was comparable to the results of Finkenstedt et al.,^29^ demonstrating the 90‐day mortality of AUCs for the MELD and MELD‐Cys were 0.90 (95% CI: 0.84–0.96) and 0.89 (95% CI: 0.84–0.94), respectively.

Some limitations occurred in our study. Firstly, this was not a community‐based study because patients were hospitalized at a referral center. Second, the study enrolled a small sample size. Third, the factors influencing mortality, such as duration of hospitalization and event of HRS, were not analyzed. Lastly, we did not validate the model to predict the risk of 90‐day mortality in an external cohort. However, our study could confirm the effectiveness of using Cys among patients with cirrhosis. Future research may quantify a combination of Cys with other novel biomarkers in a large population‐based study to identify the early detection of renal dysfunction among patients with cirrhosis. Assessing the predictors of Cys for 28‐day mortality will be recommended in further study.

## Conclusion

Serum Cys is superior to the MELD score, and the new MELD‐Cys model is as good as MELD score in predicting mortality among patients with cirrhosis admitted with complications.
